# Alkaloids in *Chelidonium majus* L: a review of its phytochemistry, pharmacology and toxicology

**DOI:** 10.3389/fphar.2024.1440979

**Published:** 2024-08-22

**Authors:** Xin-Lan Li, Yan-Ping Sun, Meng Wang, Zhi-Bin Wang, Hai-Xue Kuang

**Affiliations:** Key Laboratory of Basic and Application Research of Beiyao, Ministry of Education, Heilongjiang University of Chinese Medicine, Harbin, China

**Keywords:** *Chelidonium majus* L., alkaloids, phytochemistry, pharmacological effect, toxicity

## Abstract

*Chelidonium majus* L. (*C. majus)*, commonly known as “Bai Qu Cai” in China, belongs to the genus *Chelidonium* of the Papaveraceae family. It has rich medicinal value, such as alleviating coughs, asthma, spasms and pain. Recent studies have demonstrated that *C. majus* is abundant in various alkaloids, which are the primary components of *C. majus* and have a range of pharmacological effects, including anti-microbial, anti-inflammatory, anti-viral, and anti-tumor effects. So far, 94 alkaloids have been isolated from *C. majus*, including benzophenanthridine, protoberberine, aporphine, protopine and other types of alkaloids. This paper aims to review the research progress in phytochemistry, pharmacology and toxicology of *C. majus* alkaloids, in order to provide a theoretical basis for the application of *C. majus* in the field of medicinal chemistry and to afford reference for further research and development efforts.

## Graphical Abstract

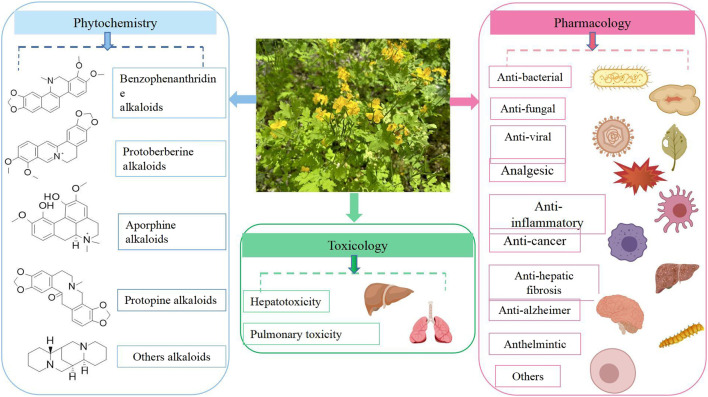

## 1 Introduction

**FIGURE 1:**
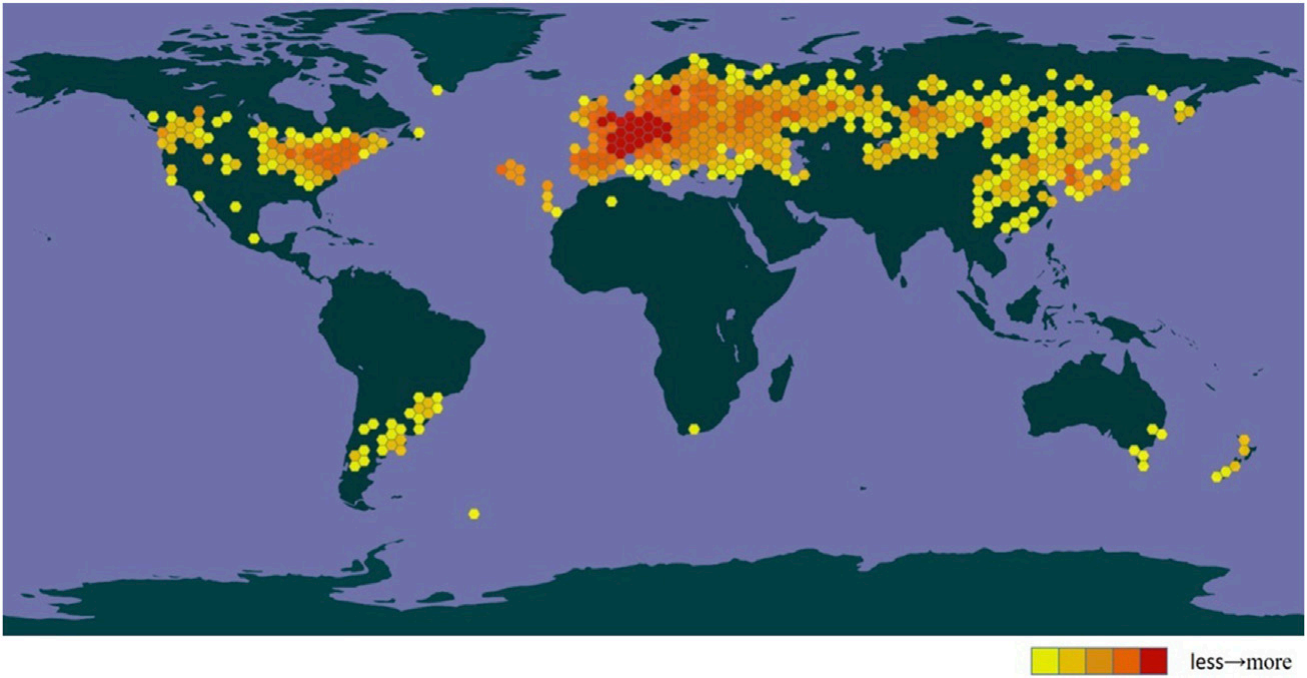
The global distributions of *Chelidonium majus* L. (https://www.gbif.org/species).

*Chelidonium majus* L., a traditional medicinal plant from the *Chelidonium* genus of the Papaveraceae family, is a perennial herb extensively distributed in Europe, Asia, and Africa (Figure 1). It has been widely used as a traditional Chinese ethnic medicine for centuries and was first documented in the “Herbal for Relief of Famines.” Known by various names in folklore, such as great celandine, swallow-wort, rock poppy, bai qu cai, tuhuanglian (土黄连) and dunchangcao (断肠草), it mostly thrives on hillsides, valley forest edges, grasslands, roadsides, and rock crevices (Wei et al., 2009; Gilca et al., 2010). According to the online records of China’s flora (http://www.cn-flora.ac.cn/index.html), the height of *C. majus* is approximately 30–60 (−100) cm. The stems are erect, multi-branched, and the branches are frequently covered with small hairs and may exude yellow latex when broken. The leaf blade is obovate-oblong or broadly obovate, 8–20 cm long. They are pinnatisect and divided into 2–4 pairs of lobes with irregularly parted or lobed crenate margins, appearing glaucous abaxially and green adaxially. Additionally, the blade is sparsely pubescent abaxially and glabrous adaxially. Flower buds are oval, with a diameter of 5–8 mm. The sepals are also oval and cymbiform, with a length of 5–8 mm. They may be glabrous or sparsely pubescent, and tend to be caducous. Petals are yellow, obovate, and entire, approximately 1 cm long. The capsule is narrowly terete at 2–5 cm × 2-3 mm, with a pedicel usually shorter than the fruit. The seeds are dark brown, ovoid, about 1 mm long or shorter, and have a shiny, alveolate appearance. The flowering and fruiting period is from April to September.

In traditional Chinese medicine (TCM), *C. majus* is classified as a heat-clearing herb (Gilca et al., 2010). The 2020 edition of the Pharmacopoeia of the People’s Republic of China describes *C. majus* as bitter, cool, and toxic, returning to the lung and stomach meridians. The whole herb has the effect of relieving spasms and pain, coughs, and asthma. As extraction techniques have evolved and advanced, significant progress in research on *C. majus* has been made. Various active ingredients of *C. majus* have been isolated and purified, including alkaloids, flavonoids, saponins, volatile oils, vitamin C, and other components (Kwasniewski, 1958; Colombo and Bosisio, 1996; Bai and Zhang, 2009). Pharmacological studies have shown that its active components exhibit wide-ranging pharmacological activities, such as antibacterial, antifungal, anti-inflammatory, antiviral, and antitumor effects (Hong et al., 2022). Further studies have identified that the major active compounds of *C. majus* are isoquinoline alkaloids (Colombo and Bosisio, 1996), including benzophenanthridine, protoberberine, aporphine, protopine, and other types (Tomè and Colombo, 1995). As a hemicryptophyte, the concentration of alkaloids in *C. majus* continues to accumulate with changes in light (Tomè and Colombo, 1995). These alkaloids are found in concentrations ranging from 0.27% to 2.25% in aerial parts and 3%–4% in the roots (Maji and Banerji, 2015), with varying alkaloid content across different plant organs. Compared to the aerial parts and underground parts, the total content of alkaloids in the leaves is lower, while the content in latex is 32 times higher than in leaves and 9 times higher than in roots (Tomè and Colombo, 1995). These findings suggest that the alkaloid content in plant organs is influenced by the number of laticifers where they are stored (Zielińska et al., 2018). Despite extensive literature reviews, we have not yet found any article that provides a comprehensive and detailed review of the alkaloids of *C. majus*. Therefore, this paper focuses on the alkaloids of *C. majus,* reviewing research progress in phytochemistry, pharmacological effects, and toxicology. The aim is to provide a reference for the application of *C. majus* in medicinal drugs, which is extremely significant for the further advancement of traditional ethnic medicine.

## 2 Methodology

To comprehensively understand the research status of *C. majus*, we conducted a thorough literature search using various electronic databases, including Web of Science, PubMed, Google Scholar, and China National Knowledge Infrastructure (CNKI). Additionally, we referred to other literature sources, such as Pharmacopoeia of the People’s Republic of China, to obtain relevant information about the alkaloids in *C. majus*. This article exclusively utilizes Chinese and English texts. The keywords employed were *C. majus* L., alkaloids, phytochemistry, pharmacological effects, and toxicity. As of May 2024, a total of 915 relevant literature sources were retrieved. To ensure the accuracy and relevance of the review, we conducted screening based on the title, abstract, and full text of the article. Duplicate articles, conference abstracts, and unavailable articles have been excluded. Additionally, articles with research purposes not relevant to the topic of this review, as well as non-English and non-Chinese articles, have also been excluded. Finally, 166 eligible articles were included.

## 3 Phytochemistry

**FIGURE 2:**
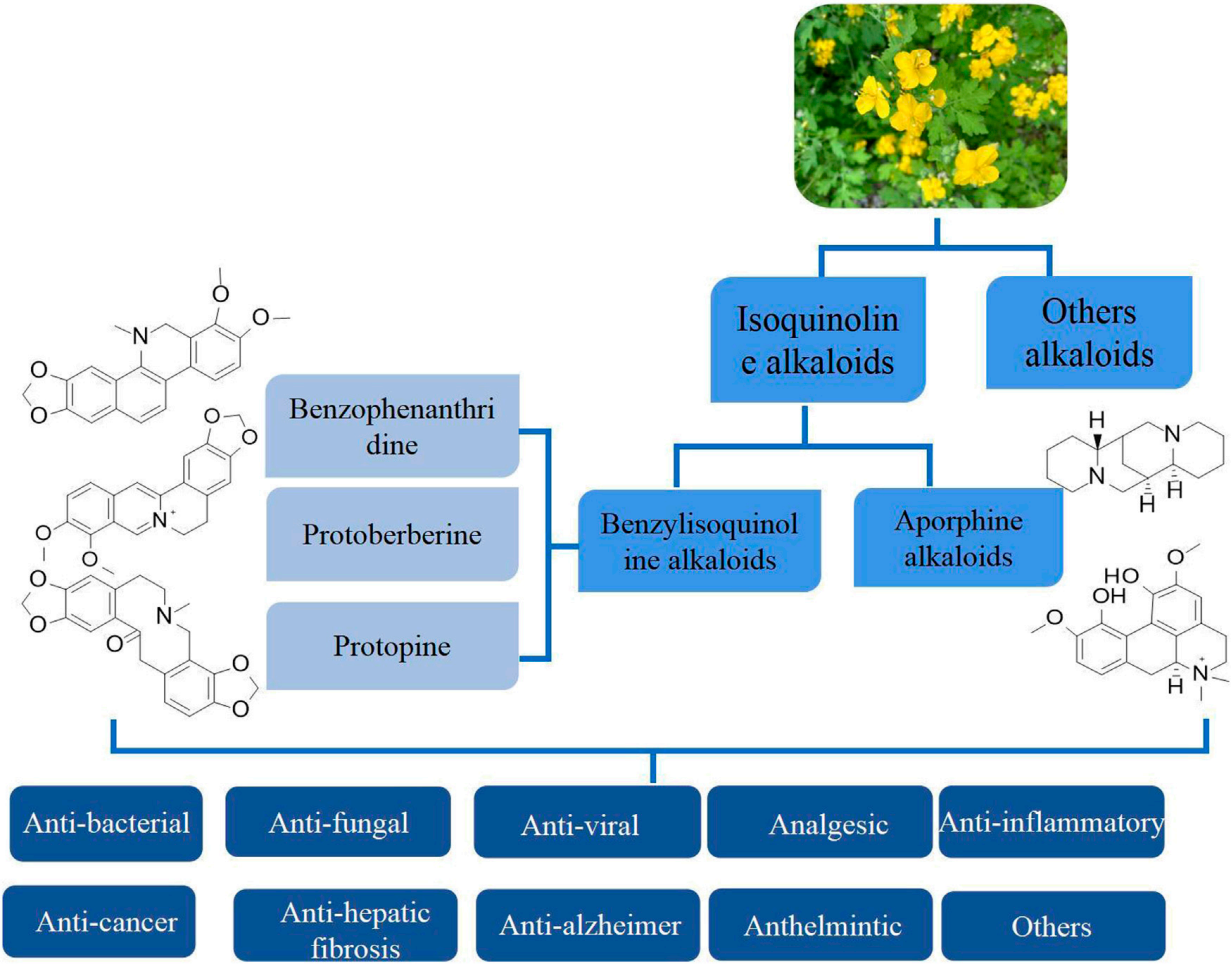
The alkaloids groups of alkaloids and pharmacological effects of *Chelidonium majus* L.

The chemical composition of *C. majus* is complex, with isoquinoline alkaloids being recognized as the main active ingredients. In addition, some scholars have reported that *C. majus* also contains flavonoids, triterpenoids, volatile oils, and other components. Isoquinoline alkaloids are a class of alkaloids derived from phenylalanine or tyrosine, which are abundant in quantity and complex in structure, providing a rich material basis for the pharmacological effects of *C. majus*. At present, 94 alkaloids have been isolated and identified from *C. majus*, which can be categorized into benzophenanthridines, protoberberines, aporphines, protopines, and other alkaloids based on their carbon skeletons (Figure 2) (Gerenčer et al., 2006; Wei et al., 2009). Among them, the three main alkaloid groups, including benzophenanthridines, protoberberines, and protopines, belong to benzylisoquinoline alkaloids, and aporphines belong to isoquinoline alkaloids, which are considered to be the active ingredients of *C. majus* and exhibit significant pharmacological activity (Tuzimski and Petruczynik, 2023; Zwerger et al., 2024). The concentrations of these alkaloids differ according to the plant parts and growth conditions, but they generally have high medicinal value. This section provides information on the types, molecular formulas, plant parts, and references of these alkaloids isolated from *C. majus*.

### 3.1 Benzophenanthridine alkaloids

**FIGURE 3:**
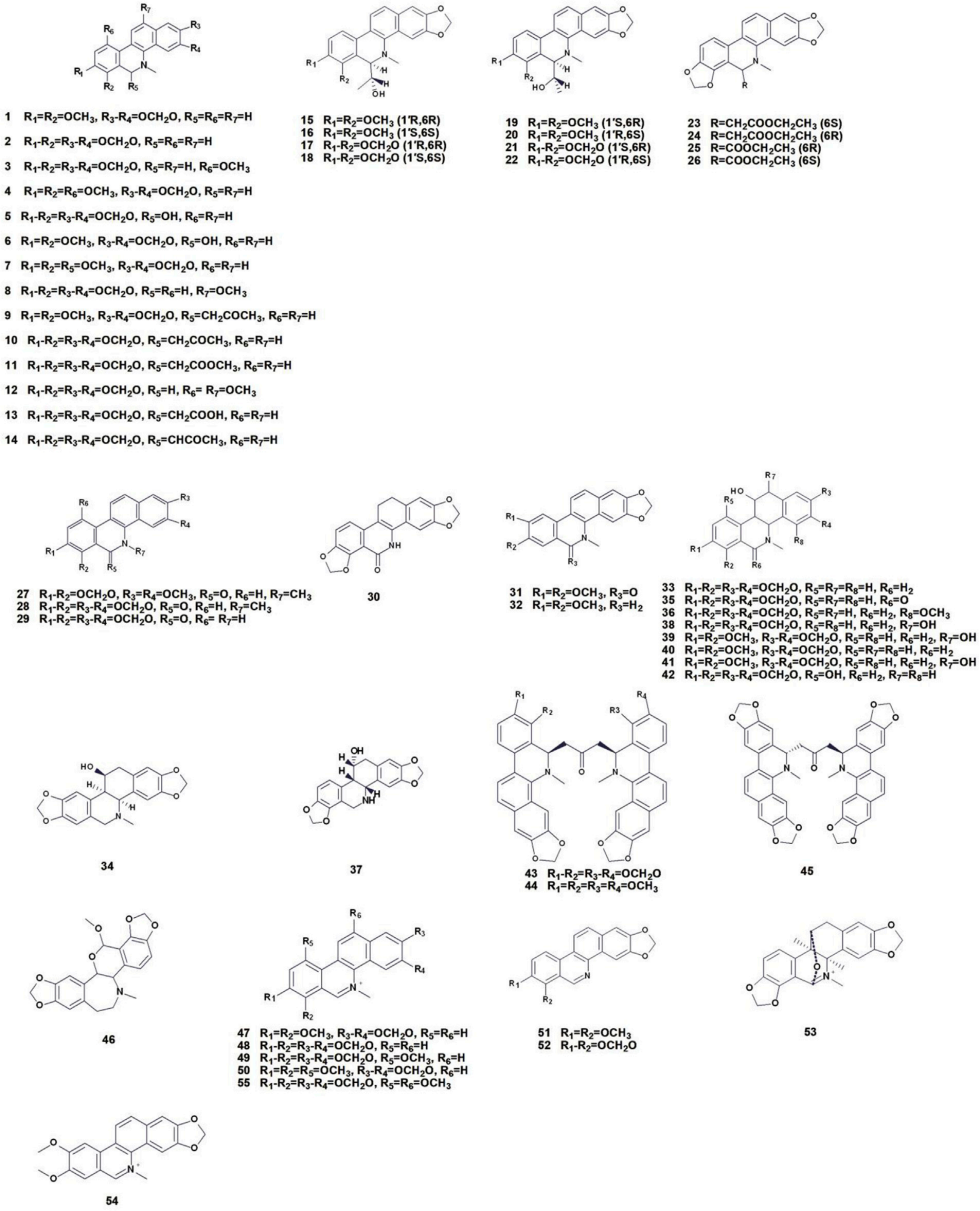
Chemical structures of benzophenanthridine alkaloids isolated from *Chelidonium majus* L. (The numbers in Figure 3 refer to the numbers of alkaloids present in Table 1).

Benzophenanthridine alkaloids are classified as isoquinoline alkaloids, characterized by a tetracyclic structural motif, and represent an important category of nitrogen-containing small molecules (Bisai et al., 2019). These alkaloids are a common in the Papaveraceae family and are the most abundant and important components in *C. majus*. Currently, 55 alkaloids (1–55) have been identified and extracted from *C. majus*. The skeletal structure consists of one isoquinoline nucleus and two benzene rings. The benzophenanthridine alkaloids isolated and identified from *C. majus* can be further divided into four structural types according to the degree of unsaturated skeleton: dihydrobenzophenthridine, hexahydrobenzophenthridine, dimindihydrobenzophenthridine and benzophenanthrine quaternary amine (Table 1; Figure 3) (Wei et al., 2009; Bisai et al., 2019; Laines-Hidalgo et al., 2022). These alkaloids are structurally variable due to their N-atom content and have been reported to have a wide range of pharmacological activities (Wei et al., 2021), with significant anti-inflammatory, analgesic, and antitumor activities (Han et al., 2016).

**TABLE 1:** Benzophenanthridine alkaloids compounds isolated from *Chelidonium majus* L.

No.	Compound name	Formula	Part of the plant	Ref.
Dihydrobenzophen-anthridine alkaloids				
1	Dihydrochelerythrine	C_21_H_19_NO_4_	Whole plant	Kadan et al. (1990), Oechslin et al. (1991)
2	Dihydrosanguinarine	C_20_H_15_NO_4_	Whole plant	Slavík and Slavíková (1977), Kadan et al. (1990)
3	Dihydrochelirubine	C_21_H_17_NO_5_	Root	Tanahashi and Zenk (1990), Táborská et al. (1994)
4	Dihydrochelilutine	C_22_H_21_NO_5_	Aerial part	Hanaoka et al. (1991), Deng et al. (2016)
5	8-Hydroxydihydrosanguinarine	C_20_H_15_NO_5_	Aerial part	Zuo et al. (2008)
6	8-Hydroxydihydrochelerythrine	C_21_H_19_NO_5_	Aerial part	Zuo et al. (2008)
7	6-Methoxydihydrochelerythine/Angoline	C_22_H_21_NO_5_	Whole plant	Zhou et al. (1989)
8	6-Methoxydihydrosanguinarine	C_21_H_17_NO_5_	Whole plant	Zhou et al. (1989)
9	8-Acetonyldihydrochelerythrine	C_24_H_23_NO_5_	Whole plant	Kadan et al. (1990)
10	8-Acetonyldihydrosanguinarine	C_23_H_19_NO_5_	Whole plant	Kadan et al. (1990)
11	Methyl-2'-(7,8-dihydrosanguinarine-8-yl) acetate	C_23_H_19_NO_6_		Park et al. (2011)
12	Dihydromacarpine	C_22_H_19_NO_6_	Aerial part	Tanahashi and Zenk (1990), Deng et al. (2016)
13	Spallidamine	C_18_H_19_NO_4_	Aerial part	Kim et al. (2015a)
14	6-Ketenesanguinarine	C_23_H_17_NO_5_	Aerial part	Zhang et al. (2014)
15	(1′R,6 R) -1-(Dihydrochelerythrine-6-yl) ethanol	C_23_H_24_NO_5_	Aerial part	Deng et al. (2017)
16	(1′S,6 S) -1-(Dihydrochelerythrine-6-yl) ethanol	C_23_H_24_NO_5_	Aerial part	Deng et al. (2017)
17	(1′R,6 R) -1-(Dihydrosanguinarine-6-yl) ethanol	C_22_H_20_NO_5_	Aerial part	Deng et al. (2017)
18	(1′S,6 S) -1-(Dihydrosanguinarine-6-yl) ethanol	C_22_H_20_NO_5_	Aerial part	Deng et al. (2017)
19	(1′S,6 R) -1-(Dihydrochelerythrine-6-yl) ethanol	C_23_H_24_NO_5_	Aerial part	Deng et al. (2017)
20	(1′R,6 S) -1-(Dihydrochelerythrine-6-yl) ethanol	C_23_H_24_NO_5_	Aerial part	Deng et al. (2017)
21	(1′S,6 R) -1-(Dihydrosanguinarine-6-yl) ethanol	C_22_H_20_NO_5_	Aerial part	Deng et al. (2017)
22	(1′R,6 S) -1-(Dihydrosanguinarine-6-yl) ethanol	C_22_H_20_NO_5_	Aerial part	Deng et al. (2017)
23	(6 S) -Ethyl 2-(dihydrosanguinarine-6-yl) acetate	C_24_H_22_NO_6_	Aerial part	Deng et al. (2017)
24	(6 R) -Ethyl 2-(dihydrosanguinarine-6-yl) acetate	C_24_H_22_NO_6_	Aerial part	Deng et al. (2017)
25	(6 R) -Ethyl-dihydrosanguinarine-6-carboxylate	C_23_H_20_NO_6_	Aerial part	Deng et al. (2017)
26	(6 S) -Ethyl dihydrosanguinarine-6-carboxylate	C_23_H_20_NO_6_	Aerial part	Deng et al. (2017)
27	Oxychelerythrine	C_21_H_17_NO_5_	Root	Táborská et al. (1994)
28	Oxysanguinarine	C_20_H_13_NO_5_	Whole plant	Kadan et al. (1990), Táborská et al. (1994)
29	N-Demethyloxysanguinarine	C_19_H_11_NO_5_		Chang et al. (2003), Wei et al. (2009)
30	N-dimethyl-9,10-dihydroxysanguinarine	C_19_H_13_NO_5_	Root	Wei et al. (2009), Kopyt’ko et al. (2005)
31	Oxynitidine	C_21_H_17_NO_5_		Maji and Banerji (2015)
32	Dihydronitidine	C_21_H_19_NO_4_	Root	Maji and Banerji (2015), Kopyt’ko et al. (2005)
Hexahydrobenzophenthridinealkaloids				
33	Chelidonine	C_20_H_19_NO_5_	Whole plant, latex	Bugatti et al. (1987), Zhao et al. (2020), Warowicka et al. (2021)
34	Isochelidonine	C_20_H_19_NO_5_	Aerial part	Rosa and Vincenzo (1992)
35	Oxychleidonine	C_20_H_17_NO_6_	Root	Kaczmarek and Malek (1959)
36	Methoxychelidonine	C_21_H_21_NO_6_	Root	Kaczmarek and Malek (1959)
37	Norchelidonine	C_19_H_17_NO_5_	Whole plant	Kadan et al. (1992)
38	Chelamine	C_20_H_19_NO_6_	Whole plant	Slavík and Slavíková (1977), Táborská et al. (1994)
39	Chelamidine	C_21_H_23_NO_6_	Whole plant	Slavík and Slavíková (1977), Táborská et al. (1994)
40	(+)-Homochelidonine	C_21_H_23_NO_5_	Whole plant	Kadan et al. (1990)
41	10-Hydroxyhomochelidonine	C_21_H_24_NO_6_	Root	Slavík and Slavíková (1977)
42	10-Hydroxychelidonine	C_20_H_19_NO_6_	Root	Slavík and Slavíková (1977)
Dimindihydrobenz-ophenthridine alkaloids				
43	Chelidimerine	C_43_H_32_N_2_O_9_	Whole plant	Tin-wa et al. (1972b), Kadan et al. (1990)
44	Chelerythridimerine	C_45_H_40_N_2_O_9_		MacLean et al. (1969), Wei et al. (2009)
45	Sanguidimerine	C_45_H_40_N_2_O_9_		Tin-wa et al. (1972a), Wei et al. (2009)
46	Rhoeadine	C_21_H_21_NO_6_	Root	Pfeifer et al. (1965), Yang et al. (2024b)
Benzophenanthrine quaternary amine alkaloids				
47	Chelerythrine	C_21_H_18_NO_4_^+^	Whole plant, latex	Bugatti et al. (1987), Warowicka et al. (2019), Zhao et al. (2020)
48	Sanguinarine	C_20_H_14_NO_4_^+^	Whole plant, latex	Kaczmarek and Malek (1959), Warowicka et al. (2019), Zhao et al. (2020)
49	Chelirubine	C_21_H_16_NO_5_^+^	Root	Kaczmarek and Malek (1959)
50	Chelilutine	C_22_H_21_NO_5_	Root	Slavík and Slavíková (1977), Táborská et al. (1994)
51	Demethylchelerythrine	C_20_H_15_NO_4_	Aerial part	Zhang et al. (2014)
52	Demethylsanguinarine	C_19_H_11_NO_4_	Aerial part	Zhang et al. (2014)
53	Didehydrochelidonine	C_20_H_20_NO_5_		Maji and Banerji (2015)
54	Nitidine	C_21_H_18_NO_4_^+^		Maji and Banerji (2015)
55	Macarpine	C_22_H_18_NO_6_^+^	Root	Táborská et al. (1994), Maji and Banerji (2015)

### 3.2 Protoberberine alkaloids

**FIGURE 4:**
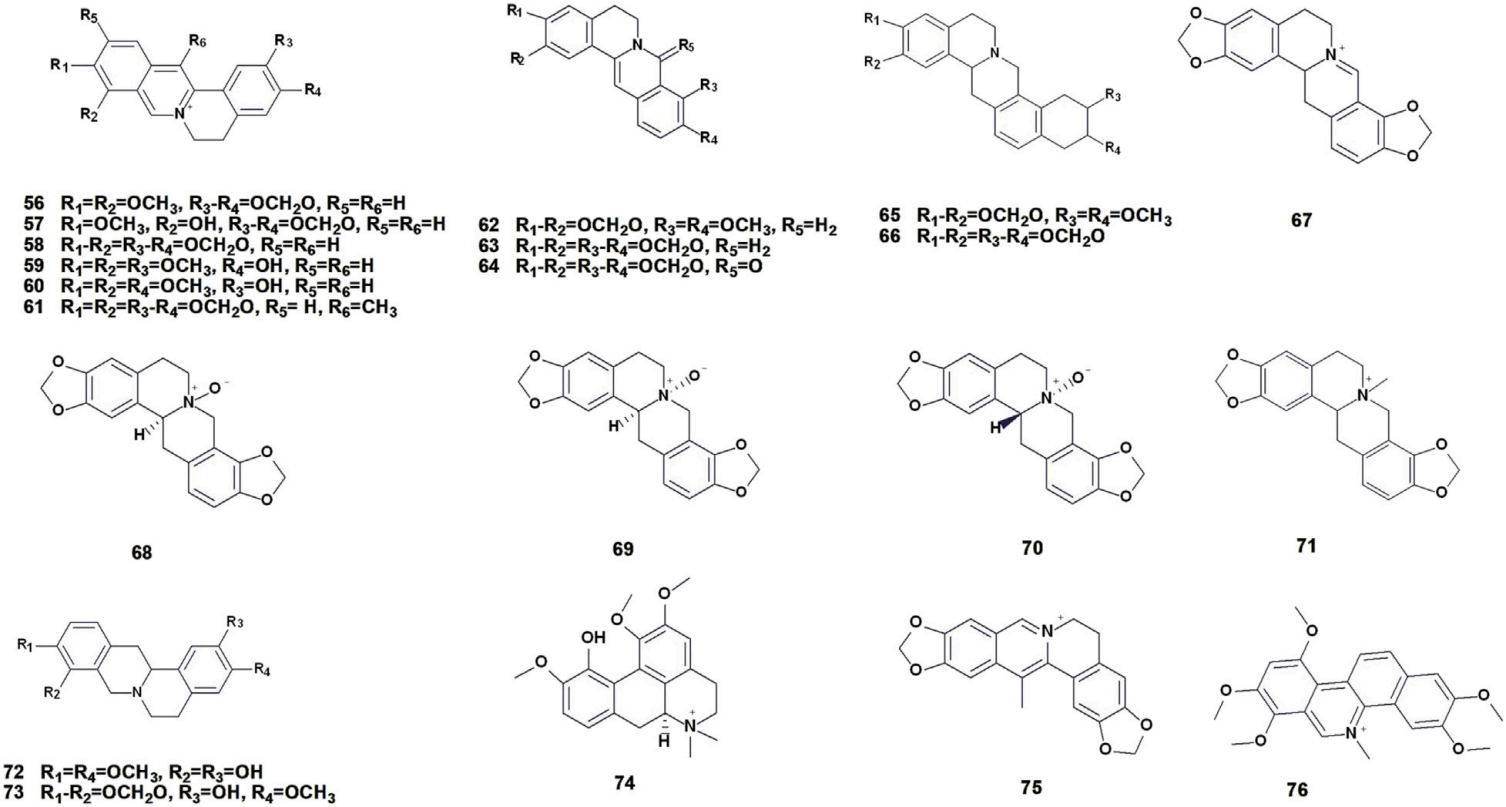
Chemical structures of protoberberine alkaloids isolated from *Chelidonium majus* L. (The numbers in Figure 4 refer to the numbers of alkaloids present in Table 2).

Protoberberine alkaloids are widely distributed and represent one of the largest categories of isoquinoline alkaloids. They are synthesized in plants through a series of complex enzymatic reactions using tyrosine as a substrate (Liu et al., 2023). This type of alkaloid is composed of two fused isoquinoline rings, primarily in the form of hydrochloride. Protoberberine alkaloids are abundantly present in nature, with *C. majus* containing a relatively high content of these compounds. Currently, 21 protoberberine compounds (56–76) have been isolated from this plant (Table 2; Figure 4). In clinical practice, this class of alkaloids demonstrates a range of biological activities, including antimicrobial and anti-inflammatory properties. These alkaloids exhibit various beneficial effects in the field of medicine.

**TABLE 2:** Protoberberine alkaloids compounds isolated from *Chelidonium majus* L.

No.	Compound name	Formula	Part of the plant	Ref.
56	Berberine	C_20_H_18_NO_4_^+^	Whole plant, latex	Bugatti et al. (1987), Zhao et al. (2020), Warowicka et al. (2021)
57	Berberrubine	C_19_H_16_NO_4_^+^	Whole plant	Yang et al. (2017), Yang et al. (2024b)
58	Coptisine	C_19_H_14_NO_4_^+^	Whole plant, latex	Sárközi et al. (2006), Zhao et al. (2020), Warowicka et al. (2021)
59	Jatrorrhizine	C_20_H_20_NO_4_^+^	Whole plant	Yang et al. (2017), Yang et al. (2024b)
60	Columbamine	C_20_H_20_NO_4_^+^	Whole plant	Yang et al. (2017), Yang et al. (2024b)
61	Corysamine	C_20_H_16_NO_4_^+^	Whole plant	Golkiewicz and Gadzikowska (1999), Zhao et al. (2020)
62	Dihydroberberine	C_20_H_19_NO_4_	Root	Kopyt’ko et al. (2005)
63	Dihydrocoptisine	C_19_H_15_NO_4_	Root	Kopyt’ko et al. (2005)
64	8-Oxycoptisine	C_19_H_13_NO_5_	Whole plant	Zhou et al. (1989)
65	Tetrahydroberberine/Canadine	C_20_H_21_NO_4_	Aerial part	Ruizhi et al. (1985), Bozhadze et al. (2013)
66	Stylopine/Tetrahydrocoptisine	C_19_H_17_NO_4_	Whole plant	Kadan et al. (1990), Sárközi et al. (2006)
67	13,14-Dihydrocoptisine	C_19_H_16_NO_4_^+^	Whole plant	Paulsen et al. (2015), Yang et al. (2024b)
68	Tetrahydrocoptisine N-oxide	C_19_H_18_NO_6_	Aerial part	Huang et al. (2019)
69	7 R,14 S-cis-tetrahydrocoptisine N-oxide	C_19_H_17_NO_5_	Aerial part	Le et al. (2021)
70	7 R,14 R-trans-tetrahydrocoptisine N-oxide	C_19_H_17_NO_5_	Aerial part	Le et al. (2021)
71	(S)-N-Methylstylopine	C_20_H_20_NO_4_^+^	Whole plant	Zhao et al. (2020)
72	Scoulerine	C_19_H_21_NO_4_	Root, leaf	Schrittwieser et al. (2011), Yahyazadeh et al. (2017)
73	Cheilanthifoline	C_19_H_19_NO_4_	Root, leaf	Yahyazadeh et al. (2017)
74	Menisperine	C_21_H_26_NO_4_^+^	Root	Tomita and Kikuchi (1955), Yang et al. (2024b)
75	Worenine	C_20_H_16_NO_4_^+^	Root	Zhang et al. (2011a), Yang et al. (2024b)
76	Sanguilutine	C_23_H_24_NO_5_^+^	Root	Táborská et al. (1994)

### 3.3 Aporphine alkaloids

**FIGURE 5:**
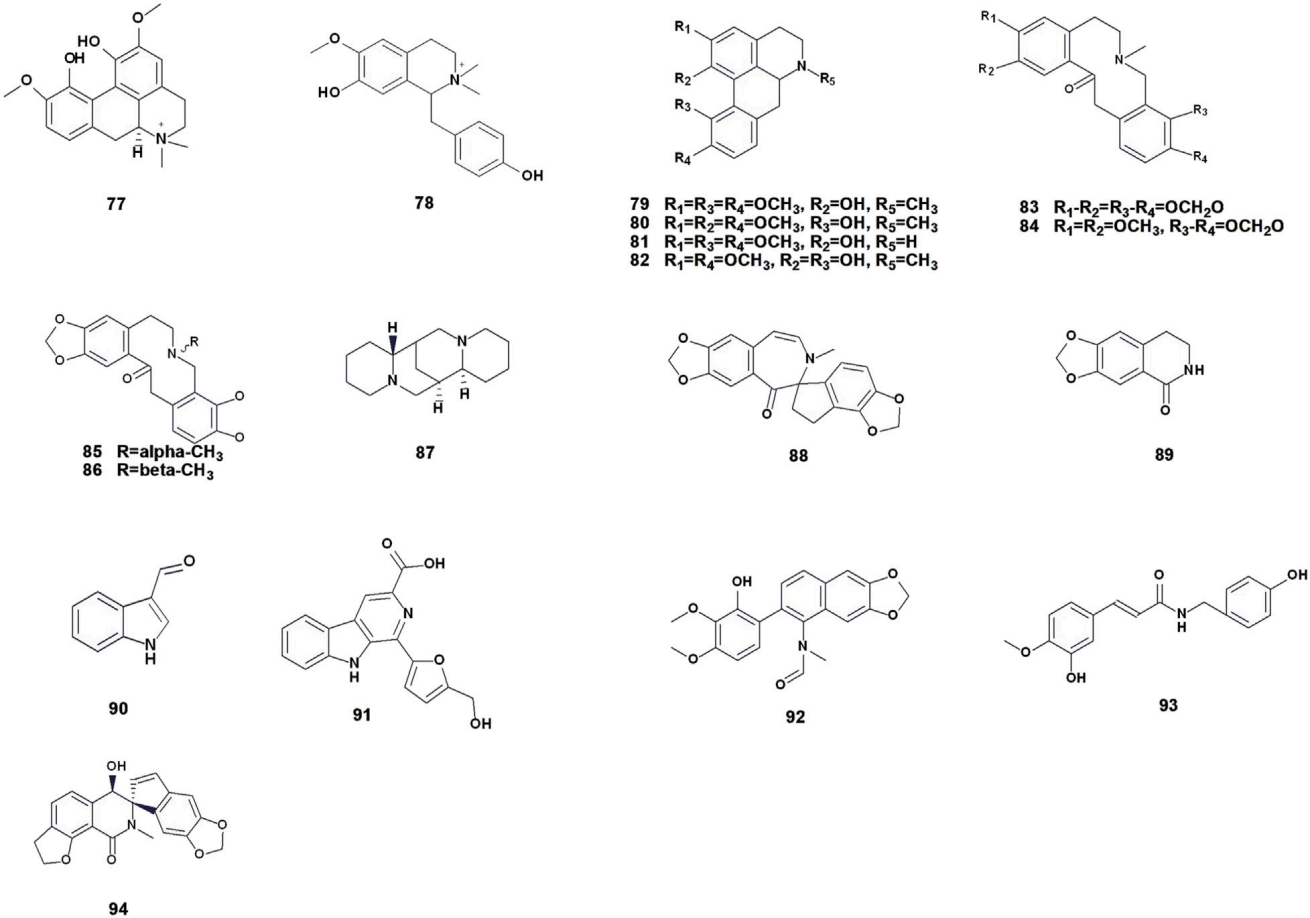
Chemical structures of aporphine, protopine and other alkaloids isolated from *Chelidonium majus* L. (The numbers in Figure 5 refer to the numbers of alkaloids present in Table 3).

Aporphine alkaloids are natural compounds that are widely distributed in nature and have important biological activities. This group of alkaloids belongs to isoquinoline alkaloids, which is an important type of natural alkaloids (Lin et al., 2020). These alkaloids are composed of four fused hexagonal rings, formed by connecting the C-2 position of the benzyl part of the benzylisoquinoline and the C-8 position of the isoquinoline part and eliminating one hydrogen molecule. A total of 6 species of aporphine alkaloids have been isolated and identified (Table 3; Figure 5). Aporphine alkaloids exhibit various pharmacological effects, including antioxidant, antiviral, and antitumor activities.

**TABLE 3:** Aporphine, protopine and other alkaloids compounds isolated from *Chelidonium majus* L.

No.	Compound name	Formula	Part of the plant	Ref.
Aporphine alkaloids				
77	Magnoflorine	C_20_H_24_NO_4_^+^	Root	Slavík and Slavíková (1977)
78	Magnocurarine	C_19_H_24_NO_3_^+^	Whole plant	Yang et al. (2017), Yang et al. (2024b)
79	Corydine	C_20_H_23_NO_4_	Root	Shafiee and Jafarabadi (1998)
80	Isocorydine	C_20_H_23_NO_4_	Root	Jeong and Lim (2017), Yang et al. (2024b)
81	Norcorydine	C_19_H_21_NO_4_	Root	Shafiee and Jafarabadi (1998)
82	Corytuberine	C_19_H_21_NO_4_	Aerial part	Táborská et al. (1994)
Protopine alkaloids				
83	Protopine	C_20_H_19_NO_5_	Whole plant	Kadan et al. (1990), Kim et al. (1999)
84	Cryptopine	C_21_H_23_NO_5_	Whole plant	Seger et al. (2004), Zhao et al. (2020)
85	α-Allocryptopine	C_21_H_23_NO_5_	Root	Kaczmarek and Malek (1959), Marek et al. (1998)
86	β-Allocryptopine	C_21_H_23_NO_5_	Root	Kadan et al. (1990), Marek et al. (1998)
Others alkaloids				
87	Sparteine	C_15_H_26_N_2_	Seed, Root, Aerial part	Kaczmarek and Malek (1959), Kopyt’ko et al. (2005)
88	(−)-Turkiyenine	C_20_H_15_NO_6_	Whole plant	Kadan et al. (1990)
89	Noroxyhydrastinine	C_10_H_9_NO_3_	Whole plant	Yuan et al. (2022)
90	Indole-3-carboxaldehyde	C_9_H_7_NO	Whole plant	Yuan et al. (2022)
91	Flazin	C_17_H_12_N_2_O_4_	Whole plant	Yuan et al. (2022)
92	Arnottianamide	C_21_H_19_NO_6_	Aerial part	Kim et al. (2015a)
93	N-Trans-feruloyltyramine	C_18_H_19_NO_4_	Aerial part	Kim et al. (2015a)
94	Chelidoniumine	C_20_H_15_NO_6_	Aerial part	Huang et al. (2019)

### 3.4 Protopine alkaloids

Protopine alkaloids are a class of isoquinoline alkaloids with ten-membered nitrogen heterocycles formed by protoberberine alkaloids through N- methylation and ring splitting in the biosynthetic pathway. The most noticeable characteristic of these compounds is C-14 carbonylation. These natural isoquinoline alkaloids possess a fundamental three-ring structure, comprising two benzene rings (A and C rings) and one ten-membered nitrogen heterocyclic ring (B ring) (Paul and Maurer, 2003). Currently, 4 species of protopine alkaloids have been isolated from *C. majus* (83–86) (Table 3; Figure 5).

### 3.5 Other alkaloids

In addition to the above four groups of alkaloids, other alkaloids have been reported. For example, Kaczmarek and Malek isolated sparteine by paper chromatography (Kaczmarek and Malek, 1959); Kadan G et al. isolated (−)-turkiyenine from the dried whole plant (Kadan et al., 1990); Yuan et al. obtained noroxyhydrastinine, indole-3-carboxaldehyde, and flazin (Yuan et al., 2022); Kim et al. identified N-trans-feruloyltyramine and arnottianamide (Kim et al., 2015); and Huang et al. extracted chelidoniumine from the aerial part of *C. majus*. Although the concentrations of these alkaloids are low, they may have unique pharmacological activities that merit further research and development (Table 3; Figure 5).

## 4 Pharmacology

To date, *C. majus* has demonstrated a diverse range of pharmacological effects, with its aerial parts and roots being rich in various alkaloids. This paper explores the alkaloids of *C. majus*, which are widely found in numerous plants and significantly impact human health. As research on *C. majus* deepens, its alkaloid components have been found to interact with multiple biological targets, exerting therapeutic effects on various diseases. These include antibacterial, antifungal, anti-tumor, anti-inflammatory, analgesic, expectorant, anti-cough, anti-asthma, and anti-liver fibrosis activities.

### 4.1 Anti-bacterial effect

Studies conducted in the past few years have shown that the alkaloids in *C. majus* have broad-spectrum antibacterial effects. Chelerythrine (CHE), extracted from *C. majus*, exhibits a strong antibacterial effect on *Streptococcus mutans*, the main caries-causing bacterium in the oral cavity. It effectively reduces the adhesion ability of *S. mutans*, suggesting its potential use as a preventative treatment for dental caries (Chen et al., 2011). *Staphylococcus aureus* (*S*. *aureus*) and *methicillin-resistant S. aureus* (MRSA) are common clinical pathogens. 8-hydroxydihydrosanguinarine (HHS) and 8-hydroxydihydrochelerythrine (HHC), solated from *C. majus*, have shown significant inhibition against MRSA strains, with minimal inhibitory concentrations/minimal bactericidal concentrations (MIC/MBC) of MRSA strains ranging from 0.49–15.63/1.95–62.50 μg/mL (Zuo et al., 2008). Sanguinarine (SNG) disrupts the cytoplasmic membrane, causing cell lysis, and is effective against MRSA, with MIC values between 3.12 μg/mL and 1.56 μg/mL, and the activity range was found to be between 3.12 μg/mL and 6.25 μg/mL (Obiang-Obounou et al., 2011). Remarkably, a study confirmed that SNG, CHE, and their derivatives exhibit robust antibacterial effects against *S. aureus*, *Escherichia coli* (*E. coli*), and *Aeromonas hydrophila* (Miao et al., 2011). Another study evaluated the antimicrobial potential of the major alkaloids in *C. majus* and found that *C. majus* was most effective against *Pseudomonas aeruginosa* (MIC of 1.9 mg/L), while SNG showed effectiveness against *S. aureus* (MIC of 1.9 mg/L) (Zielińska et al., 2019). Moreover, chelidonine (CHLD), SNG, and CHE also demonstrated inhibitory effects on *E. coli* (Móricz et al., 2015). These antibacterial effects have been summarized in Table 4.

**TABLE 4:** The anti-bacterial and anti-fungal effects of alkaloids from *Chelidonium majus* L.

Compounds	Models	Positive control	Results	Ref.
Anti-bacterial				
Chelerythrine	*Streptococcus* mutans		Exhibited inhibitory activity	Chen et al. (2011)
	*Pseudomonas aeruginosa*		MIC = 1.9 mg/L	Zielińska et al. (2019)
	*S. aureus*, *E. coli*, *Aeromonas* hydrophila	Penicillin sodium Ceftriaxone sodium	Exhibited inhibitory activity	Miao et al. (2011)
Sanguinarine	*S. aureus*, *E. coli*, *Aeromonas* hydrophila	Penicillin sodium Ceftriaxone sodium	Exhibited inhibitory activity	Miao et al. (2011)
Sanguinarine	*S. aureus*		MIC = 1.9 mg/L	Zielińska et al. (2019)
Sanguinarine	MRSA	Ampicillin Ciprofloxacin	MIC = 3.12–6.25 μg/mL	Obiang-Obounou et al. (2011)
Sanguinarine	*E. coli*		Exhibited inhibitory activity	Móricz et al. (2015)
Chelidonine	*E. coli*		Exhibited inhibitory activity	Móricz et al. (2015)
HHS	MRSA	Vancomycin	MIC/MBC = 0.49–15.63 μg/mL	Zuo et al. (2008)
HHC	MRSA	Vancomycin	MIC/MBC = 1.95–62.50 μg/mL	Zuo et al. (2008)
Anti-fungal				
Chelerythrine	*Candida* albicans	Penicillin sodium	MIC = 2–16 μg/mL	Gong et al. (2019)
	Ustilaginoidea virens		EC_50_ = 6.53 × 10^−3^ mg/mL	Wei et al. (2020)
	Cochliobolus miyabeanus		EC_50_ = 5.62 × 10^−3^ mg/mL	Wei et al. (2020)

### 4.2 Anti-fungal effect

The alkaloids and derivatives in *C. majus* exhibit significant antifungal activity, and the mechanism of action has gradually been revealed. This provides innovative insights and approaches for the development of new antifungal agents and the control of agricultural diseases. CHE induces the accumulation of reactive oxygen species (ROS) by increasing the intracellular calcium concentration in mycelium, thus inhibiting the growth of *Candida albicans* mycelium, with MIC values ranging from 2 to 16 μg/mL (Gong et al., 2019). Furthermore, Wei QH’s experiment studied the *in vitro* antifungal activity of CHE against five rice pathogenic fungi. The results indicated that the EC_50_ values for *Ustilaginoidea virens* and *Cochliobolus miyabeanus* were 6.53 × 10^−3^ mg/mL and 5.62 × 10^−3^ mg/mL, respectively (Wei et al., 2020). These antifungal effects have been summarized in Table 4.

### 4.3 Anti-viral effect

The yellow latex of *C. majus* is widely used in folk medicine to treat human papillomavirus (HPV) because of its antiviral properties. Recent studies have found that its antiviral effect is mainly attributed to the alkaloid and protein components contained in the latex, which can target different stages of the virus replication cycle, effectively reducing HPV infection, and suppressing the expression of the viral oncogenes (E6, E7) at the mRNA and protein levels (Musidlak et al., 2022). Moreover, CHE can directly target the gB and gD glycoproteins on the surface of HSV-1, thereby inhibiting HSV-1 infection by preventing the binding of the virus to cells (Hu et al., 2023). In addition to its effect on human viruses, *C. majus* can also be employed for the prevention and control of agricultural plant virus es. Three alkaloids isolated from *C. majus* have shown **activity against** Tobacco mosaic virus (TMV). CHE and CHLD significantly inhibit TMV, while SNG moderately reduces TMV infection, thereby mitigating virus-induced damage in plants (Guo et al., 2021).

### 4.4 Analgesic effect

The alkaloids in *C. majus* exhibit significant analgesic effects on inflammatory pain, cancer-related pain, peripheral neuralgia and other types of pain, demonstrating high clinical application value. At present, *C. majus* is used as the main ingredient in clinical analgesic drugs. For example, Weitongshu capsules alleviate pain associated with gastric ulcers, and the compound Chinese medicine Tongan injection treats cancer pain caused by radiotherapy and chemotherapy or non-radiotherapy and chemotherapy. The analgesic mechanism of *C. majus* extract is different from that of morphine, indicating that it does not act as a narcotic analgesic and is devoid of side effects such as addiction, showing peripheral analgesic effects that are highly valued in clinical application (Li et al., 2013). CHE is one of the primary constituents of *C. majus*, and progress has been made in understanding its analgesic mechanism. It mitigates the occurrence of neuropathic pain by inhibiting the activation of PKC and spinal cord astrocytes (Chen et al., 2014). In addition, for chronic pain such as functional abdominal pain, a combination of network pharmacology and molecular docking technology revealed that alkaloids in *C. majus* mainly induce central analgesia through a network mode of multiple target interventions, targeting SRC, AKT1, EGFR, CASP3, and MAPK3, resulting in anti-functional abdominal pain effects (Zhang et al., 2023).

### 4.5 Anti-inflammatory effect

Inflammation is common in clinical practice, making the study of anti-inflammatory drugs essential. CHE can inhibit the production of PGE2 by modulating COX-2, a crucial enzyme in the response to inflammation, thus exerting an anti-inflammatory effect (Niu et al., 2011). The tumor necrosis factor (TNF)-induced nuclear factor-kappa B (NF-κB) signaling pathway has been discovered in some types of inflammation, among which the TNF-α/NF-κB pathway is a well-studied typical inflammatory signaling pathway and the core of coordinating inflammatory immune response. CHE was found to mitigate the inflammatory response of lipopolysaccharide (LPS)-induced serum levels of TNF-α and NO production in mouse models of endotoxin shock (Li et al., 2012). Another study found that CHE protects against LPS-induced acute lung injury, inhibits the production of inflammatory factors such as TNF-α, IL-6, and IL-1β, and reduces pulmonary edema and neutrophil infiltration. The mechanism may be related to the inhibition of NF-κB activation and interference with the nuclear translocation of Nrf2 protein (Fan et al., 2018). CHE also significantly reduces the gastric ulcer index, inhibits NO concentration, IL-6 and TNF-α levels in serum and gastric mucosa of mice with gastric ulcers, while markedly attenuating the overexpression of NF-κB in the gastric mucosa to exert anti-inflammatory activity (Li et al., 2014). CHE is used to treat mice with acetic acid-induced ulcerative colitis, an inflammatory bowel illness, by blocking the generation of NO and TNF-α inflammatory cytokines (Xu et al., 2014; Wu et al., 2022). It has also been found that CHE can promote apoptosis and autophagy in rheumatoid arthritis by influencing the expression of genes related to autophagy and apoptosis (including Bax, Bcl-2, PARP, and ULK1) and the AMPK/mTOR/ULK-1 signaling pathway, thus inhibiting rheumatoid arthritis *in vivo* and *in vitro* (Cai et al., 2022). In addition, it regulates key signaling pathways in SARS-CoV-2 infection (including Nrf2, NF-κB, and p38 MAPK activity) to prevent excessive inflammatory immune responses (Valipour et al., 2021).

Several researchers have also isolated 6-acetonyl-5,6-dihydrosanguinarine (ADS) from *C. majus* and discovered that ADS can induce the production of the inflammatory cytokines TNF-α, IL-6, and IL-8 by macrophages and dendritic cells. These inflammatory cytokines are important for the inflammatory response, and their excessive production often leads to the aggravation of inflammation and the development of diseases. ADS can trigger the release of pro-inflammatory cytokines through the ROS-JNK/ERK-NF-κB signaling pathway, thus inhibiting the occurrence of the inflammatory response (Kim et al., 2013).

Furthermore, CHLD exhibits significant anti-inflammatory actions, inhibiting LPS-induced inflammatory responses *in vitro* and *in vivo* by blocking the TLR4/NF-κB signaling pathway in RAW264.7 macrophages (Liao et al., 2018). It has been experimentally demonstrated that inhibiting TNF-induced NF-κB activation and modulating NF-κB regulatory gene products display anti-inflammatory, anti-proliferative, pro-apoptotic, and anti-invasion effects. Therefore, CHLD can alleviate the inflammatory response in HCT 116 human colon cancer cells (Zhang et al., 2018). CHLD inhibits IL-1β-mediated inflammation by modulating the NF-κB pathway *in vitro*, thereby preventing cartilage degeneration and synovial inflammation in rats with osteoarthritis (Li et al., 2023). In particular, CHLD also suppresses the production of IL-4, IL-17, eotaxin-2, and Ovalbumin-specific IgE through the STAT6 and FOXP3 pathways and can be used to treat airway inflammation (Kim et al., 2015). CHLD can prevent inflammatory damage in porcine small intestinal epithelial cell line IPEC-J2 cells by significantly reducing pro-inflammatory factors and promoting IL-10 expression (Lin et al., 2024).

In addition to the above two alkaloids, there may be other ingredients in *C. majus* with anti-inflammatory activities. These components might influence the inflammatory response through different mechanisms, thereby synergistically enhancing the anti-inflammatory effects of *C. majus.* The anti-inflammatory actions of these alkaloids were examined in macrophage RAW264.7 cells to ascertain their inhibitory impact on the production of NO caused by LPS. CHLD and HHS exhibited strong inhibitory activities on NO production in LPS-induced macrophage RAW 264.7 cells (Park et al., 2011). The impact of five alkaloids on the secretion of IL-1β, IL-8, and TNF-α in human polymorphonuclear leukocytes (neutrophils) was determined. It was found that berberine, CHLD, and CHE notably reduced TNF-α secretion in a concentration-dependent manner, while SNG inhibited IL-1β secretion and coptisine slightly decreased TNF-α, IL-1β, and IL-8 secretion (Zielińska et al., 2020). In another study, stylopine was found to inhibit macrophage NO in a concentration-dependent manner by suppressing the expression of iNOS, COX-2, NO, and PGE2, which may be related to the anti-inflammatory activities of *C. majus* (Jang et al., 2004).

### 4.6 Anti-cancer effect

Cancer is currently the primary cause of disease-related death in humans, and both its incidence and mortality are rising globally (Chen et al., 2024). Therefore, research and treatment of cancer are particularly urgent. Researchers both domestically and overseas have carried out a significant number of *in vitro* and *in vivo* experiments on the anti-tumor effects of *C. majus* in recent years. Studies have indicated that alkaloids extracted and isolated from *C. majus* have significant biological activities in anti-tumor treatment, inhibiting the proliferation, migration, and invasion of tumor cells through various mechanisms. These alkaloids play an anti-cancer role in cervical cancer, lung cancer, liver cancer, and other cancers by promoting apoptosis, altering the cell cycle, inducing autophagy, and activating mitochondrial apoptosis. These anti-cancer effects have been summarized in Table 5.

**TABLE 5:** The anti-cancer effects of alkaloids from *Chelidonium majus* L.

Compounds	Models	Results	*In Vivo* */* *In Vitro*	Ref.
Chelidonine	HepG2 cells	Inhibited the proliferation	*In vitro*	Noureini and Wink (2009)
	MHCC97-H cells, LM3 cells, nude mice or BalB/c mice	Inhibited the process of EMT and enhances the antitumor effect of lenvatinib on HCC cells, IC_50_ = 7.72 ± 0.70 μmol/L and 6.34 ± 0.44 μmol/L, respectively	*In vivo* and *in vitro*	Hou et al. (2019)
	H1975 cells, nude mice	Inhibited cell growth *in vitro* and *in vivo*, IC_50_ = 2.58 ± 1.05 μmol/L	*In vivo* and *in vitro*	Xie et al. (2020)
	SGC-7901 cells	Induced mitotic slippage and apoptotic-like death, IC_50_ = 23.13 μmol/L		Qu et al. (2016)
	BALB/c mice, Renca C	Effectively inhibited tumor proliferation	*In vivo*	Pan et al. (2023)
	KB cells	Inhibited proliferation, invasion and promoted apoptosis	*In vitro*	Tao and Ran (2018)
	BxPC-3, MIA PaCa-2 cells	Induced apoptosis	*In vitro*	Jang et al. (2021)
	MCF-7 cells	Strongly suppressed cell growth	*In vitro*	Noureini and Esmaili (2014)
	MDA-MB-231 cells	Inhibited migration and invasion of cells	*In vitro*	Kim et al. (2015b)
	T98G cells	Through multipolar spindle assembly causing G2/M arrest in T98G cells	*In vitro*	Lee et al. (2019)
Chelerythrine chloride	SMMC-7721 cells	Inhibited tumor growth and induced apoptosis	*In vitro*	Zhang et al. (2011b)
	MHCC97-H cells	Inhibited cell growth, invasion and migration	*In vitro*	Cheng et al. (2019)
	HEK-293 and SW-839 cells	Inhibited cell proliferation and induced apoptosis	*In vitro*	Chen et al. (2016)
Chelerythrine	HepG2 cells	IC_50_ of 6,12,24 h was 12.98, 10.53, 11.21 μmol/L, respectively	*In vitro*	Han and Zhu (2016)
	HepG2 cells	Induced apoptosis	*In vitro*	Lin et al. (2022)
	C57BL/6 mice	Inhibited tumor growth	*In vivo*	Jin and Lu (2024)
	BGC823 cells	IC_50_ of 24, 48, 72 h was 2.87, 0.903, 0.468 μg/mL, respectively	*In vitro*	Zong and Liu (2006)
	HeLa cells	Induced apoptosis	*In vitro*	Yu et al. (2000)
	SKOV3 cells	Inhibited the proliferation, migration, invasion, and EMT	*In vitro*	Zhou et al. (2024)
	HL-60 cells	Induced apoptosis and necrosis, IC_50_ = 2.6 μmol/L	*In vitro*	Vrba et al. (2008)
	B16 cells	Inhibited proliferation and induced apoptosis	*In vitro*	Zhang et al. (2018a)
	5–8 F cells	Inhibited cell proliferation and induced apoptosis	*In vitro*	Chen et al. (2024)
	HCT-116, RKO cells	Inhibited cell growth and induced apoptosis	*In vitro*	Liu and Jiang (2019)
	Zebrafish, ACC2 cells	Inhibited cell growth and proliferation and induced apoptosis	*In vivo* and *in vitro*	Li et al. (2021a)
Sanguinarine	HeLa cells	Induced apoptosis	*In vitro*	Alakkal et al. (2022)
	SKOV3 cells	Inhibited cell viability, promoted cell apoptosis and suppressed cell migration and invasion	*In vitro*	Zhang et al. (2018b)

#### 4.6.1 Liver cancer

Isoquinoline alkaloids in *C. majus* have anti-telomerase activity, a key target of therapeutic intervention in cancer cells. Experiments have demonstrated that CHLD inhibits telomerase activity in HepG2 cells by down-regulating the expression of human telomerase reverse transcriptase and inducing apoptosis (Noureini and Wink, 2009; Noureini et al., 2017). CHLD inhibits the epithelial-mesenchymal transition (EMT) process, enhances the apoptotic effect of lenvatinib on HCC cells in nude mice, and reduces the *in vivo* growth of hepatocellular carcinoma tumors (Hou et al., 2019). Another study indicated that chelerythrine chloride (CHECL) significantly inhibits SMMC-7721 cell proliferation in a time-and dose-dependent manner by blocking the S-phase of SMMC-7721 cells, and activating the mitochondrial apoptosis pathway by regulating the expression of Bcl-2 family proteins, thus inducing apoptosis in SMMC-7721 cells (Zhang et al., 2011). CHECL also inhibits growth, invasion, and migration in the highly metastatic human hepatocellular carcinoma cell line MHCC97-H (Cheng et al., 2019). Additionally, CHE upregulates the relative expression of Bax and Caspase-3 proteins and mRNA, decreases the relative expression of Bcl-X_L_ protein and mRNA, prevents proliferation in HepG2 hepatoma cells, and ultimately induces apoptosis (Han and Zhu, 2016). Experiments by Lin’s team showed that CHE exposure induces excess ROS generation and triggers oxidative stress and mitochondrial apoptosis pathways in HepG2 cells, ultimately causing apoptosis in HepG2 cells (Lin et al., 2022).

#### 4.6.2 Lung cancer

Lung cancer (LC) is a serious health problem that can lead to significant morbidity and mortality. In the treatment of LC, CHE can reduce tumor growth in Lewis lung cancer transplanted mice by targeting the NF-κB/HIF-1α signaling pathway and down-regulating the expression levels of NF-κB and HIF-1α proteins, thereby achieving a therapeutic effect (Jin and Lu, 2024). Clinical findings indicate that the majority of non-small cell lung cancer (NSCLC) patients exhibit mutations in the epidermal growth factor receptor tyrosine kinase (EGFR), leading to increased EGFR activity and facilitating metastasis and progression of NSCLC (Jiang et al., 2022). Targeted therapy directed at mutant forms of EGFR, such as the small molecule inhibitor Gefitinib, has shown successful application in the treatment of NSCLC patients. Clinical data supports its efficacy in significantly prolonging patient survival; however, prolonged use often leads to acquired resistance in most individuals (Bai et al., 2019). Therefore, researchers found that CHLD can inhibit the growth of Gefitinib-resistant non-small cell LC cells by modulating the EGFR-AMPK signaling pathway. They also found that CHLD mediates apoptosis through the mitochondrial pathway by decreasing the expression of AKT and Bcl-2 and increasing the cleavage of Bax and Caspase-3 expression (Xie et al., 2020).

#### 4.6.3 Gastric cancer

Gastric cancer is a malignant neoplasm originating in the upper lining of the stomach. CHE can effectively induce apoptosis in human gastric cancer (BGC 823) cells, and this apoptosis is cell cycle-dependent (Zong and Liu, 2006). In another study, CHECL was found to inhibit cell proliferation in a time - and dose-dependent manner, causing cell cycle arrest and, apoptosis in BGC-823 cells through the reduction of mitochondrial membrane potential, release of cytochrome c, activation of caspase-3, disruption of PARP, and dysregulation of BCL-2 family proteins (Zhang et al., 2012). Additionally, CHLD can induce SFC-7901 M phase arrest and mitotic slippage in human gastric cancer cells by down-regulating the expression of BubR1, Cdk1 and cyclin B1 proteins (Qu et al., 2016).

#### 4.6.4 Renal cell carcinoma

Renal cell carcinoma is among the ten most prevalent cancers in humans. CHLD has been found to regulate the expression of proteins Smad3 and Smad7 in the TGF-β1/Smad pathway to inhibit tumor proliferation in tumor-bearing mice with renal cell carcinoma (Pan et al., 2023). Chen et al. (2016) also demonstrated that CHECL may induce apoptosis in renal cancer cells by inhibiting ERK activity.

#### 4.6.5 Breast cancer

Breast cancer (BC) is a disease that threatens human life and health worldwide. According to TCM, BC belongs to the category of “ru yong” and “ru shi yong," mainly caused by the deficiency of zheng qi and the imbalance of yin and yang in the viscera (Liu et al., 2021). CHLD is highly cytotoxic to cancer cells and induces MCF-7 BC cell death by potently inhibiting telomerase activity and stimulating multiple mechanisms of cell death, including apoptosis, autophagy, and senescence (Noureini and Esmaili, 2014). In addition, CHLD demonstrates anti-migration and anti-invasion effects in MDA-MB-231 BC cells by preventing the formation of the integrin-linked kinase/PINCH/α-parvin complex (Kim et al., 2015). CHE can play an anti-BC role through the PI3K/AKT signaling pathway (Zhang et al., 2021). CHLD inhibits the mitosis of BC cells by inducing M-phase arrest and blocking the AKT/FOXO3/FOXM1 axis, thus exerting anti-BC effects (Li et al., 2024). Chelidonium Herba-Corydalis Rhizoma is one of the commonly used prescriptions for BC in TCM. It can suppress the expression of ERα, p-PI3K, p-Akt protein, and ESR1 mRNA, as well as inhibit the growth of MCF-7 cells (IC_50_ value: 693 μg/mL), suggesting that its anti-ER-positive BC effect may be connected to the modulation of ER and PI3K/Akt signaling pathways (Zou et al., 2023).

#### 4.6.6 Cervical cancer

CHE has been proven to exert anti-tumor effects by inducing apoptosis in HeLa cells through activation of the P38/JNK signaling pathway (Yu et al., 2000). Chelerythrine hydrochloride inhibits the proliferation of cervical cancer HeLa cells by triggering mitochondrial apoptosis via the PI3K/BAD signaling pathway (Yang et al., 2020). Another isoquinoline alkaloid, CHLD, has also shown effectiveness in triggering apoptosis in HeLa cells, exerting anti-cancer effects by upregulating the expression of pro-apoptotic genes such as, p38 and p53 and downregulating the expression of anti-apoptotic genes including AKT, PI3K, JAK3, STAT3, E6, and E7 (Paul et al., 2012). Protoberberine alkaloids (coptisine, berberine, and their derivatives, like stylopine) isolated from *C. majus* have demonstrated anti-cervical cancer activity by interfering with reactive oxygen species production, intracellular caspase activation, and mitochondrial function (Warowicka et al., 2019). The main latex proteins in *C. majus*, along with berberine, 8-hydroxycheleritrine, and dihydroberberine, can reduce the *in vitro* activity of human cervical cancer cells (both HPV-negative and HPV-positive) and synergistically play an anti-cancer role (Nawrot et al., 2021). SNG has long been considered an anti-tumor drug; studies have shown that it induces death in HeLa cells by activating apoptosis and ferroptosis (Alakkal et al., 2022).

#### 4.6.7 Ovarian cancer

Ovarian cancer, often termed the “silent killer” due to its high mortality rate, shows promising response to SNG in epithelial ovarian cancer cells by controlling the CASC2-EIF4A3 axis, blocking NF-κB signaling, or the PI3K/AKT/mTOR pathway (Zhang et al., 2018). Meanwhile, research on CHE indicates its effectiveness in inhibiting proliferation, migration, and invasion of human Ovarian cancer SKOV3 cells, preventing or alleviating the occurrence of SKOV3 cell epithelial-mesenchymal transition, and suppressing tumor metastasis (Zhou et al., 2024).

#### 4.6.8 Leukemia

CHE and dihydrochelerythrine have been shown to arrest the cell cycle of HL-60 cells in the G1 phase, alter cell cycle distribution, and activate the mitochondrial apoptosis pathway, inducing apoptosis and necrosis in human leukemia HL-60 cells (Vrba et al., 2008). Studies have also indicated that CHE and SNG induce dose-dependent DNA damage and increased cytotoxicity in primary mouse spleen cells and mouse Lymphpocytic Leukemia L1210 cells, while CHLD does not exhibit significant cytotoxic or DNA damaging effects on these cells but can completely inhibit the growth of L1210 cells (Kaminskyy et al., 2008). Moreover, the Havelek R team confirmed significant cytotoxicity of CHLD and homochelidonine, effectively inducing leukemia cell death (Havelek et al., 2016). SNG, berberine, and *C. majus* extracts have also exhibited significant cytotoxic and pro-apoptotic activities against hematopoietic cell lines HL-60, HL-60/MX1, HL-60/MX2, CCRF/CEM and CEM/C1, J45.01, and U266B, suggesting their potential utility in treating various types of leukemia (Och et al., 2019).

#### 4.6.9 Melanoma

Melanoma, a type of skin cancer originating from the malignant transformation of melanocytes, is concerning due to its malignancy and treatment challenges. CHE inhibits the proliferation of B16 cells in a dose- and time-dependent manner significantly increases the early and late apoptosis rates of B16 cells, and upregulates the expression levels of Caspase-3 and Bax genes, while reducing the expression levels of Bcl-2 genes. It has been demonstrated that CHE suppresses the activation of the Wnt/β-catenin signaling pathway, thereby slowing B16 cell proliferation and promoting apoptosis (Zhang et al., 2018). Experimental data have shown that uveal melanoma cells undergo necrotic cell death and apoptosis when exposed to benzophenanthine alkaloids (SNG, CHE, and CHLD) (Kemény-Beke et al., 2006). Furthermore, benzphenanthridine alkaloids, including chelilutine, CHE, and SNG, exhibit a strong antiproliferative effect on malignant melanoma cells; these alkaloids induce apoptosis by reducing levels of anti-apoptotic proteins (Bcl-xL, Mcl-1, and xIAP), leading to decreased mitochondrial membrane potential and cleavage of caspase-3 and PARP (Hammerová et al., 2011).

#### 4.6.10 Nasopharyngeal carcinoma

SNG, in combination with 5-fluorouracil, synergistically inhibits the growth of nasopharyngeal carcinoma grafts *in vivo*, inducing autophagy and apoptosis related to the PI3K/AKT/mTOR signaling pathway (Peng et al., 2022). SNG inhibits the growth of human nasopharyngeal carcinoma 5–8 F cells, induces autophagy, and suppresses proliferation by activating AMPK/mTOR signaling (Su et al., 2022). Experiments confirm that both SNG and CHE inhibit nasopharyngeal carcinoma cell proliferation and induce apoptosis by regulating the PI3K/AKT and MAPK signaling pathways (Chen et al., 2024).

#### 4.6.11 Others

CHLD increases the growth of the human oral epithelioid cancer cell line KB in a time- and dose-dependent manner, and inhibits KB cells invasion in a dose-dependent manner. It upregulates Bax expression and decreases Bcl-2 expression, activating Caspase-3 and inducing KB cell apoptosis. This effect may be mediated through dual inhibition of Akt and MAPK signaling pathways, inhibiting Bcl-2 expression and promoting Caspase-3 expression, thereby suppressing cancer cell proliferation, fostering apoptosis, and impeding tumor growth and metastasis (Tao and Ran, 2018). Moreover, CHLD induces G2/M phase block in BxPC-3 and MIA PaCa-2 cells by downregulating CDK 1, and increases S-phase block induced by GADD 45a by upregulating p21 and p53, culminating in pancreatic cancer cell apoptosis through Caspase-3 cleavage (Jang et al., 2021). Dihydrosanguinarine exhibits inhibitory effects on K-Ras and TP53 mutant pancreatic cancer cell lines by bidirectionally modulating mut-p53/-Ras and WT-p53/-Ras proteins (Wu et al., 2019). CHLD induces apoptosis in human glioblastoma cells through G2/M phase arrest and Mcl-1 degradation (Lee et al., 2019). CHE triggers apoptosis by activating ROS-mediated mitochondrial dysfunction in colorectal cancer cells (Liu and Jiang, 2019). CHE effectively inhibits the growth and proliferation of adenoid cystic carcinoma cells and induces apoptosis by increasing ROS levels and upregulating NF-κB, p-JNK and p-p38 expression in cells (Li et al., 2021).

### 4.7 Anti-hepatic fibrosis effect

The mRNA expression of TGF-1, Smad3, Smad4, and the negative regulator Smad7 varied significantly following different doses of CHE in the mouse model of carbon tetrachloride-induced hepatic fibrosis, suggesting that CHE could inhibit the signaling of the TGF-β receptor complex from the cytoplasm to the nucleus. Additionally, CHE interfered with the expression of TGF-β1, Smad4, and Smad7 proteins, further confirming its inhibitory effect on hepatic fibrosis in mice, associated with the TGF-β/Smads signaling pathway (Li et al., 2018). After administering different doses of CHLD to rats with carbon tetrachloride-induced hepatic fibrosis, the protein phosphorylation levels of PI3K, Akt, mTOR, and mRNA expression levels of corresponding genes in their tissues were increased to varying degrees. This indicates that CHLD can regulate the mRNA and protein expression of genes related to the PI3K/Akt/mTOR pathway, affecting the expression of autophagy marker proteins LC3 and p62, preventing the activation of hepatic stellate cells, and inhibiting liver fibrosis (Li et al., 2021). Using TGF-β1-activated rat hepatic stellate cells CFSC-8B as a model of hepatic fibrosis, it was observed that CHLD inhibited the proliferation of TGF-β1-activated hepatic stellate cells, further demonstrating its potential to reverse liver fibrosis (Li et al., 2019).

### 4.8 Anti-alzheimer effect

Alzheimer’s disease (AD) is a progressive neurodegenerative disease with an insidious onset that causes neuronal damage in the brain, leading to memory loss, cognitive decline, and behavioral changes. Acetylcholinesterase plays a critical role in nerve function, as it is a serine protease produced by motor neurons and muscle junctions that hydrolyzes the neurotransmitter acetylcholine into acetic acid and choline, thereby terminating nerve impulses. Decreased levels of acetylcholine are a key factor in the onset of AD. Two compounds, HHC and HHS, isolated from *C. majus*, exhibit strong inhibitory activity against acetylcholinesterase. They slow down the breakdown of acetylcholine and increase its levels in the brain. Therefore, these compounds have been suggested as potential alternatives to anti-dementia drugs (Cho et al., 2006).

### 4.9 Anthelmintic effect

*Chelidonium majus* not only holds medicinal value, but also serves as a pesticide. Plant-derived alkaloid insecticides are crucial components of plant pesticides, showing significant repellent and insecticidal activities against numerous pests. CHLD has been found to possess substantial anthelmintic activity against Dactylogyrus intermedius, achieving a 100% anthelmintic effect at 0.9 mg L^−1^. The EC_50_ value after 48 h of exposure (the concentration required to achieve a 50% deworming effect) was 0.48 mg L^−1^ (Yao et al., 2011). The insecticidal mechanisms of alkaloids in *C. majus* are diverse, contributing to larvicidal effects by disrupting enzyme activity, reducing food intake, affecting nutritional indexes, and downregulating mRNA expression of enzyme genes (Zou et al., 2017). In another study, alkaloids in *C. majus* induced resistance to dietary intake and larval mortality in *Lymantria dispar* by inhibiting food intake and digestive enzymes (Zou et al., 2019).

### 4.10 Other effects

In the isolated ileal spasmolytic test model of guinea pigs, CHLD and protopine, when their concentration reaches 1 × 10^−5^ g/mL, can significantly induce ileal relaxation in response to barium chloride stimulation, with relaxation rates reaching 68.8% and 54.8%, respectively (Hiller et al., 1998). The DPPH free radical scavenging rate was determined using the DPPH method, with ascorbic acid as a positive control to evaluate the antioxidative activity of CHE solid dispersion (SD). The results showed that different mass concentrations of CHE-PEG-SD exhibited certain DPPH radical scavenging ability (IC_50_ = 0.124 mg/mL), albeit weaker than ascorbic acid (IC_50_ = 0.041 mg/mL) (Wang et al., 2020). Traditional medicine traditions utilize natural small-molecule compounds from *C. majus* to treat various gynecological disorders, including coptisine, protopine, berberine, and dihydroberberine (Lans et al., 2018). Supplementation with the protoberberine-rich fraction from *C. majus* in the diet of rats with experimentally induced endometriosis has been shown to inhibit the recurrence of endometriosis (Warowicka et al., 2021).

## 5 Toxicology

The utilization of plants and herbs in traditional medicine has a long history, with many herbs used to treat various ailments. However, it’s crucial to remember that not all herbs are harmless. *Chelidonium majus* was recorded as a toxic Chinese medicine in the Chinese Pharmacopoeia of the People’s Republic of China (2020 edition). The alkaloids found in *C. majus*, including CHLD, SNG, berberine, coptisine, and CHE, either alone or in combination, possess potential toxicity (Benninger et al., 1999). Following evaluation using liver-targeted causality assessment methods, several cases of hepatotoxicity were found to be likely or highly likely related to *C. majus*. Therefore, the toxicity of *C. majus* to liver function has always been a topic of concern, with some components potentially causing liver damage through different mechanisms. The potential hepatotoxicity of *C. majus* has also been confirmed in several reports from European countries, defining this hepatotoxicity as a unique form of herbal-induced liver injury (HILI) due to idiosyncratic metabolic reactions (Pantano et al., 2017). Ciornolutchii et al. reported two case studies in which patients exhibited liver damage after using pharmaceutical preparations containing *C. majus*, characterized by severe hypertransaminasemia. Their literature review also identified multiple HILI cases related to *C. majus* preparations (Ciornolutchii et al., 2024). The metabolism of CHLD was studied in a human liver microsomal model, revealing two demethylated metabolites containing phenolic hydroxyl groups after incubation with liver microsomes. These hydroxyl groups are easily oxidized into quinone compounds, which can combine with glutathione to form quinone-sulfide, depleting glutathione in the liver and causing hepatotoxicity (Zhang et al., 2018).

Alkaloids, in *C. majus*, such as CHE, SNG, berberine and coptisine, have significant inhibitory effects on mitochondrial respiration in mice, suppressing liver respiration by inhibiting mitochondrial enzymes like NADH dehydrogenase or succinic acid (Barreto et al., 2003). Furthermore, SNG induces chromosome breaks and DNA damage in mouse bone marrow cells (Das et al., 2004). Additionally, CHLD, berberine, and SNG from *C. majus* distinctly block hERG potassium channels, delaying heart repolarization and prolonging QT intervals, potentially increasing the risk of death (Orvos et al., 2015). Chronic exposure to *C. majus* can also cause toxic effects in specific organs; CHE has been observed to induce a dose-dependent long-term toxicity in rat lung tissue, resulting in symptoms of pulmonary congestion and bloody ascites (Liu et al., 2019). Despite *C. majus* having abundant pharmacological effects, caution should be exercised to avoid excessive or prolonged use to prevent toxic reactions.

Chinese herbal medicines can be processed to reduce or eliminate drug toxicity and side effects, while also modifying drug function and flavor, thereby improving therapeutic efficacy. Researchers have processed *C. majus* and discovered that the content of SNG and CHE in licorice products derived from *C. majus* is significantly lower than in raw products. The optimal processing method involves using 15% licorice, moistening for 3 h, and drying at 60°C for 12 h. This suggests that licorice processing technology has a certain detoxification effect (Xiao et al., 2021). In conclusion, although *C. majus* possesses numerous pharmacological effects and holds certain application value in the pharmaceutical field, its potential toxicity risks must also be given sufficient attention. A deeper understanding of its efficacy and safety through scientific research is essential, and it should be used cautiously, with a full understanding of its potential risks to prevent toxic reactions from excessive or long-term use.

## 6 Discussion

This plant has been revered for its antibacterial, antiviral, antitumor, anti-inflammatory, and other pharmacological effects for centuries. Alkaloids from C. majus, particularly benzophenanthridine and protoberberine alkaloids, show promising potential for application in anti-tumor therapy (Yang et al., 2024). Compounds such as CHE, CHLD, and SNG, known for their high content and notable anti-tumor activity, have been extensively studied. The alkaloids exert anti-tumor effects by promoting apoptosis, altering the cell cycle, inducing autophagy, and activating mitochondrial apoptosis. Even alkaloids present in small amounts, such as berberine (Mohammadlou et al., 2021), dihydrochelerythrine (Silva et al., 2018), 6-methoxydihydrosanguinarine (Wang et al., 2023), and berberrubine (He et al., 2023), demonstrate significant potential in tumor treatment. UkrainTM, a derivative of C. majus alkaloids comprising components like CHLD, CHE, SNG, protopine, and allocryptopine, induces apoptosis in cells and exerts toxic effects on cancer cells (Habermehl et al., 2006). However, the exact anti-tumor mechanisms of C. majus alkaloids remain unclear, necessitating further in-depth studies to elucidate their mechanisms of action.

*Chelidonium majus* alkaloids not only exhibit anti-tumor effects but also demonstrate significant antimicrobial effects. These alkaloid components are applied to human diseases and also play a role in preventing and controlling on plant diseases as well. Simultaneously, these alkaloids exert anti-inflammatory effects and treat various inflammations by regulating the immune system and various inflammation-related signaling pathways. As research on the pharmacological effects of C. majus alkaloids progresses, there is potential for further expanding their clinical applications. Analysis of other alkaloids with lower content has revealed that aporphine alkaloids, such as magnoflorine, may reduce blood glucose levels by promoting insulin release and stimulating insulin activity mechanisms, potentially improving postprandial hyperglycemia and demonstrating anti-diabetic effects (Patel and Mishra, 2011). Vennerstrom et al. discovered that protoberberine alkaloids showed potential antimalarial activity *in vitro* experiments (Vennerstrom and Klayman, 1988), while Xiang et al. explored the potential uses of these alkaloids in treating stomach diseases and providing gastric protective effects (Xiang et al., 2024). Berberine has also been noted to prevent and delay AD (Wang et al., 2024), while protopine may hold a potential role in asthma treatment (Yang et al., 2024). However, studies on these effects are limited and mostly at a preliminary stage. The results of pharmacological activity studies are mostly based on animal experiments, and the results of clinical studies may differ; further research is needed to verify their effects and mechanisms. Additionally, the synergistic effects between C. majus alkaloids and other drugs or ingredients cannot be overlooked and warrant further exploration.

The rich chemical composition and extensive biological activity of C. majus alkaloids provide valuable resources for the development of pharmaceutical drugs. However, there still exist limitations in the extraction process, content determination, and the relationship between structure and activity. Future research efforts should focus on optimizing extraction processes, improving the purity of bioactive alkaloids, delving into the relationship between structure and activity, elucidating the underlying mechanisms of action, and facilitating their practical application in the field of medicine.

## 7 Conclusions and perspectives

*Chelidonium majus*, a medicinal plant with a long history of application, has been extensively used in European countries as well as in China’s TCM, highlighting its extremely high medicinal value. Alkaloids are the main active components of the plant, which have garnered attention from many scholars. These alkaloids are abundant in content and variety, but each alkaloid exhibits unique chemical structures and can act on multiple biological targets to play a therapeutic role in diseases. This makes *C. majus* a vast potential area for research and development in drug research.

To date, researchers both domestically and internationally have isolated and identified 94 alkaloids from *C. majus*. This review therefore summarizes the research findings on the phytochemistry, pharmacology, and toxicology of these alkaloids. Additionally, it systematically classifies these alkaloids according to their structural properties, providing a foundational basis for the phytochemical classification of *C. majus*.

Through further in-depth study of these alkaloids, more candidate compounds can be provided for drug research and development, expanding their potential applications in the pharmaceutical field. However, it is worth noting that these alkaloids also have some toxicity. Therefore, in the application of *C. majus* for drug development or treatment of diseases, it is necessary to further explore the toxic mechanisms of these alkaloids to optimize the extraction process, reduce toxicity, and improve efficacy, thereby laying a solid foundation for their broad application in the pharmaceutical field. Simultaneously, strengthening the quality control and safety evaluation of *C. majus* is also crucial for future research directions, to effectively manage potential risks during drug development and ensure the safety and effectiveness of drugs. In summary, this review provides a basis for understanding the current research status of alkaloid components in *C. majus*. As science and technology advance and research methods improve, more comprehensive studies on *C. majus* alkaloids are expected, aiming to develop clinically valuable drugs and provide a solid theoretical framework for the in-depth research and application of *C. majus*.

## Abbreviations

*C. majus, Chelidonium majus L.;* TCM, Traditional Chinese medicine; CHE, Chelerythrine; SNG, Sanguinarine; CHLD, Chelidonine; CHECL, Chelerythrine chloride; HHS, 8-hydroxydihydrosanguinarine; HHC, 8-hydroxydihydrochelerythrine.
